# Performance of salivary microbiota in detecting periodontitis using a machine learning approach

**DOI:** 10.3389/fcimb.2025.1631798

**Published:** 2025-09-18

**Authors:** Shinya Kageyama, Shion Hama, Michiko Furuta, Mikari Asakawa, Shintaro Kawano, Toshiharu Ninomiya, Toru Takeshita

**Affiliations:** ^1^ Section of Preventive and Public Health Dentistry, Division of Oral Health, Growth and Development, Faculty of Dental Science, Kyushu University, Fukuoka, Japan; ^2^ Section of Oral and Maxillofacial Oncology, Division of Maxillofacial Diagnostic and Surgical Sciences, Faculty of Dental Science, Kyushu University, Fukuoka, Japan; ^3^ Center for Cohort Studies, Graduate School of Medical Sciences, Kyushu University, Fukuoka, Japan; ^4^ Department of Epidemiology and Public Health, Graduate School of Medical Sciences, Kyushu University, Fukuoka, Japan

**Keywords:** oral microbiota, saliva, LightGBM, SHAP, screening

## Abstract

Altered salivary microbiota due to the progression of periodontitis may serve as a marker for simple and accurate identification of periodontitis. In this study, we examined saliva samples collected from 2,050 community-dwelling adults using 16S rRNA gene sequencing and verified the predictive performance of salivary microbiota in detecting periodontitis using a light gradient boosting machine algorithm. Five-fold stratified cross-validation was applied with 10 iterations, and the predictive performance was evaluated using the mean area under the receiver operating characteristic curve (AUC) value. In detecting periodontitis defined by number of teeth with probing depth ≥4 mm, localized (≥2 teeth), intermediate (≥4 teeth), and generalized (≥6 teeth) cases were detected with mean AUC values of 0.81 (95% confidence intervals, 0.80–0.81), 0.85 (0.84–0.86), and 0.87 (0.87–0.88), showing an increasing trend with extent. According to the Shapley additive explanation analysis, *Porphromonas gingivalis*, *Tannerella forsythia*, *Mycoplasma faucium, Treponema* species HMT-237, and *Fretibacterium* species HMT-362 were identified as important features for the detection of periodontitis. Our study presents the potential of salivary microbiota as a tool for mass screening of periodontitis and provides information on novel and important targets, including taxa other than known periodontal pathogens, to establish salivary screening tests.

## 1 Introduction

Periodontitis is an inflammatory oral disease that arises from complex interactions between the host immune response and dental plaque microorganisms (Pihlstrom et al., 2005; Papapanou et al., 2018). Clinically, it is characterized by the resorption of the alveolar bone and formation of a deep periodontal pocket, eventually resulting in tooth loss. Periodontitis is also known to be associated with various systemic diseases, such as cardiovascular disease, rheumatoid arthritis, and respiratory disease (Pihlstrom et al., 2005; Hajishengallis, 2014; Angjelova et al., 2024; Dolcezza et al., 2024). Therefore, early detection and intervention in periodontitis are crucial for maintaining oral and systemic health. However, it often remains undetected until it progresses to a severe state owing to its asymptomatic nature in the early stages. Periodontal examinations by dental professionals, either dentists or hygienists, are required for the detection and diagnosis of periodontitis, which is a technical, time-consuming, and invasive process. Therefore, there is an urgent need to develop a novel approach for accurate and simple detection of periodontitis without specialized training.

Saliva is a promising specimen for the detection of periodontitis because it can be easily and noninvasively collected. Various salivary components, such as occult blood, enzymes, cytokines, and proteins, have been investigated for their potential to detect periodontal disease (Lamster et al., 2003; Nomura et al., 2006; Shimazaki et al., 2011; Maeng et al., 2016; Wu et al., 2018; Liaw et al., 2023; Lu et al., 2023). However, no definitive conclusions or methodologies have been established. In particular, we focused on salivary microbiota as a reasonable biomarker. Briefly, the progression of periodontitis increases the subgingival space of the periodontal pocket, which is occupied by obligate anaerobic and proteolytic bacteria. In parallel, salivary microbiota contains bacteria shed from the subgingival space as a minor component and their occupancy in salivary microbiota increases with the progression of periodontitis (Umeda et al., 1998; He et al., 2012; Haririan et al., 2014; Belstrøm et al., 2017; Kageyama et al., 2017; Jung et al., 2024). Considering these factors, it is reasonable to predict periodontal condition by examining the salivary microbiota. In line with these findings, we examined the salivary microbiota, focusing on subgingival bacteria, and demonstrated that it can be used for the identification of periodontitis with high predictive performance (Ma et al., 2021). However, although this performance was remarkable in generalized cases of periodontitis, it was limited in the detection of localized cases.

In this study, we used a light gradient boosting machine (LightGBM) based on salivary microbiota data to predict periodontitis. LightGBM is a high-performance machine learning algorithm based on gradient boosting decision trees designed for efficiency and scalability, enabling fast and accurate analysis of numerous variables with nonlinearity and complex feature interactions (Ke et al., 2017). In this study, we aimed to investigate the predictive performance of salivary microbiota in the detection of periodontitis, including localized cases, using this machine learning approach and to identify the key bacterial species in the prediction model.

## 2 Materials and methods

### 2.1 Study participants

The participants in this study were community-dwelling adults in Hisayama town, Japan (Hata et al., 2013). As a part of the health examination of Hisayama residents, we conducted dental examinations and saliva sampling of participants aged ≥39 years in 2012. Of the 2,654 participants who underwent dental examination, saliva samples sufficient for microbiota analysis were collected from 2,100 participants. After excluding 50 participants with <2 teeth (the required minimum for definition of outcomes, n=49) and those with missing probing depth (PD) data (n=1), 2,050 participants were finally included in the analysis. Written informed consent was obtained from all participants. The Ethics Committee of Kyushu University approved the present study and the procedure for obtaining informed consent (approval number: 23092).

### 2.2 Dental examination and saliva sample collection

Dental examinations and sample collection were conducted according to a previously described protocol (Takeshita et al., 2016). Briefly, the periodontal condition was evaluated by PD and bleeding on probing at two sites for all teeth except the third molars (mesio- and mid-buccal sites) based on the NHANES III method. Following the dental examination, we instructed the participants to chew gum for 2 min and collected their whole stimulated saliva in sterile plastic tubes. The collected saliva samples were stored at -80°C until analysis.

### 2.3 DNA extraction and 16S rRNA gene analysis

DNA was extracted from the saliva samples using the bead-beating method described previously (Kageyama et al., 2022, 2023). The V1–V2 regions of 16S rRNA gene were amplified using the following primers: 8F (5′-AGA GTT TGA TYM TGG CTC AG-3′) with the sample-specific tag sequence and 338R (5′-TGC TGC CTC CCG TAG GAG T-3′). Polymerase chain reaction amplification and purification were performed as described previously (Takeshita et al., 2016). The purified amplicons were pooled and sequenced using an Ion PGM Hi-Q Sequencing kit (Thermo Fisher Scientific) on an Ion PGM (Thermo Fisher Scientific). Quality filtering of all raw sequence reads was performed using a script manually written in R software (version 4.2.3). The reads that exhibited <200 bases, had an average quality score ≤25, or did not include the correct forward and reverse primer sequences were excluded from the analysis. The remaining reads were demultiplexed by examining the tag sequence at the forward end and the forward and reverse primer sequences were trimmed. The quality-checked reads (fastq.gz) were imported into QIIME 2 (version 2023.2.0) and directly clustered against 16S rRNA gene sequences in eHOMD (version 15.22) with a minimum identity of 97% using the vsearch cluster-features-closed-reference plugin in QIIME 2 (Chen et al., 2010; Rognes et al., 2016; Bolyen et al., 2019). Finally, an abundance table of salivary microbiota, including 802 taxa, was generated.

### 2.4 Outcomes

The severity of periodontitis was defined by the Centers for Disease Control and Prevention (CDC) and the American Academy of Periodontology (AAP) case definitions (Eke et al., 2012) or the biological definitions based on the number of teeth with PD ≥4 mm. As the biological definitions, we defined presence of ≥2, ≥4, and ≥6 teeth with PD ≥4 mm (top 5th, 10th, and 20th percentiles for number of teeth with PD ≥4 mm) as localized, intermediate, and generalized periodontitis. The outcomes of this study were mild, moderate, and severe periodontitis based on the CDC and AAP case definitions and localized, intermediate, and generalized periodontitis based on the biological definition, as binary classifications (such as severe and non-severe).

### 2.5 Machine learning analysis

**Figure 1:**
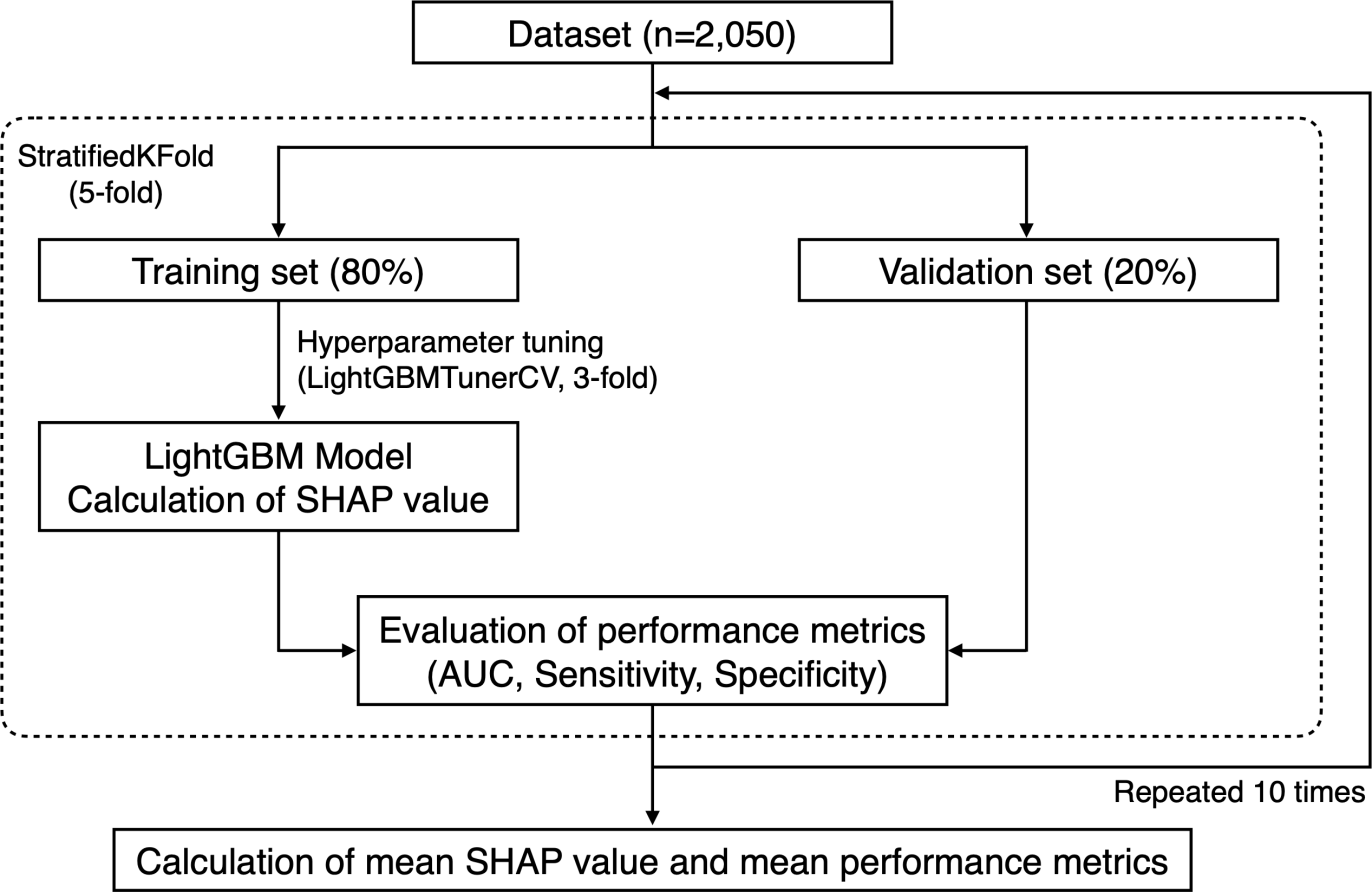
Flow chart of machine learning procedures. For testing intermediate and generalized periodontitis according to the biological definitions, 10 and 24 participants without the required minimum number of teeth for definitions were excluded, respectively.

All machine learning analyses were performed using the Python software (version 3.12.5). For testing intermediate and generalized periodontitis according to the biological definitions, 10 and 24 participants with <4 and <6 teeth (the required minimum for definitions), respectively, were excluded. To focus on the predictive performance of the salivary microbiota, the dataset was composed only of age, sex, and the relative abundance of each taxon in the salivary microbiota. We applied a five-fold stratified cross-validation using the StratifiedKFold function from the scikit-learn library (version 1.5.1) (Pedregosa et al., 2011) (**Figure 1**). Over all five-fold training/validation splits, the model was fitted to the training set using LightGBM (version 4.5.0). Performance metrics, including the area under the receiver operating characteristic curve (AUC), sensitivity, and specificity, were assessed using the validation set. The optimal cut-off values for determining sensitivity and specificity were calculated based on the Youden index, which maximizes the sum of sensitivity and specificity (Youden, 1950). This cross-validation process was iterated 10 times, and 50 values were obtained for each performance metric. Hyperparameters were primarily set as objective=binary, metric=auc, is_unbalance=True, and force_col_wise=True, and further tuned using the LightGBMTunerCV function (n_splits=3) from the Optuna library (version 4.0.0) (Akiba et al., 2019). The best parameters obtained were used to fit the model using the training set. To interpret the model, we computed the Shapley additive explanation (SHAP) values (Lundberg et al., 2020). The SHAP framework assigns each feature an importance value for prediction, enabling interpretation of the predictions of complex models. We computed the SHAP values of all the features in each trained model (50 models) fitted to the training set, and we calculated the mean of the absolute SHAP values for each feature (version 0.46.0).

## 3 Results

### 3.1 Characteristics of participants and 16S rRNA gene sequencing

We examined the salivary microbiota of 2,050 participants (934 male and 1,116 female) aged 39–90 years (median: 61 years). The median number of teeth present was 26 (interquartile range [IQR]: 22–28) and 33.9% of participants had ≥28 teeth (**Table 1**). According to the CDC and AAP case definitions, 3.8%, 18.4%, and 6.5% of the participants had mild, moderate, and severe periodontitis, respectively. Regarding the biological definitions, 12.0%, 5.2%, and 5.7% of the participants had localized, intermediate, and generalized periodontitis, respectively. Their saliva samples were analyzed using 16S rRNA gene amplicon analysis and finally 21,796,606 reads (9534.8 ± 3219.8 reads per sample) were obtained to determine the bacterial composition of salivary microbiota. The salivary microbiota of each participant comprised a median of 198 (IQR: 172–223) bacterial species and was dominated by *Rothia mucilaginosa*, *Prevotella melaninogenica*, *Neisseria subflava*, *Streptococcus salivarius*, and *Granulicatella adiacens*.

**Table 1:** Characteristics of the study participants.

Characteristic	Participants (n=2,050)
Age (years)	60.3 ± 12.0
Male	934 (45.6)
Number of teeth	24.3 ± 5.6
Number of teeth with probing depth ≥4 mm	1.2 ± 2.6
Periodontitis based on the CDC/AAP definitions	
None	1,462 (71.3)
Mild	77 (3.8)
Moderate	377 (18.4)
Severe	134 (6.5)
Periodontitis based on the biological definitions	
None	1,583 (77.2)
Localized	245 (12.0)
Intermediate	106 (5.2)
Generalized	116 (5.7)

Data are presented as mean ± standard deviation for age and number of teeth and n (%) for categorical variables. As the biological definitions, presence of ≥2, ≥4, and ≥6 teeth with probing depth ≥4 mm was defined as localized, intermediate, and generalized periodontitis.

CDC, Centers for Disease Control and Prevention; AAP, American Academy of Periodontology.

### 3.2 Predictive performance of salivary microbiota in detecting periodontitis

Prediction models were constructed using LightGBM with the bacterial composition data of the salivary microbiota. The predictive performance in detecting periodontitis according to each definition is presented in **Table 2**. The mean AUC values for detecting localized, intermediate, and generalized periodontitis defined by the biological definitions were 0.81 (95% confidence intervals [CI], 0.80–0.81), 0.85 (0.84–0.86), and 0.87 (0.87–0.88), respectively, showing an increasing trend with severity. Although severe periodontitis according to CDC and AAP case definitions were detected with an AUC value of 0.83 (0.82–0.84), the performance for detecting mild and moderate periodontitis were lower than those when using the biological definitions, with AUC values of 0.77 (0.77–0.78) and 0.78 (0.77–0.79).

**Table 2:** Predictive performance of salivary microbiota in detecting periodontitis.

Outcome	AUC	Sensitivity	Specificity
CDC/AAP definitions			
Mild	0.77 (0.77–0.78)	0.73 (0.71–0.76)	0.70 (0.68–0.72)
Moderate	0.78 (0.77–0.79)	0.74 (0.72–0.77)	0.70 (0.68–0.72)
Severe	0.83 (0.82–0.84)	0.83 (0.80–0.86)	0.73 (0.70–0.76)
Biological definitions			
Localized	0.81 (0.80–0.81)	0.76 (0.74–0.78)	0.75 (0.73–0.76)
Intermediate	0.85 (0.84–0.86)	0.83 (0.81–0.85)	0.76 (0.74–0.77)
Generalized	0.87 (0.87–0.88)	0.86 (0.84–0.89)	0.77 (0.75–0.80)

The data are presented as mean values (95% confidence intervals) on 50 iterations. As the biological definitions, presence of ≥2, ≥4, and ≥6 teeth with probing depth ≥4 mm was defined as localized, intermediate, and generalized periodontitis. Sensitivity and specificity were determined using cut-off values based on the Youden index. AUC, area under the receiver operating characteristic curve, CDC, Centers for Disease Control and Prevention; AAP, American Academy of Periodontology.

### 3.3 Important features for detecting periodontitis

**Figure 2:**
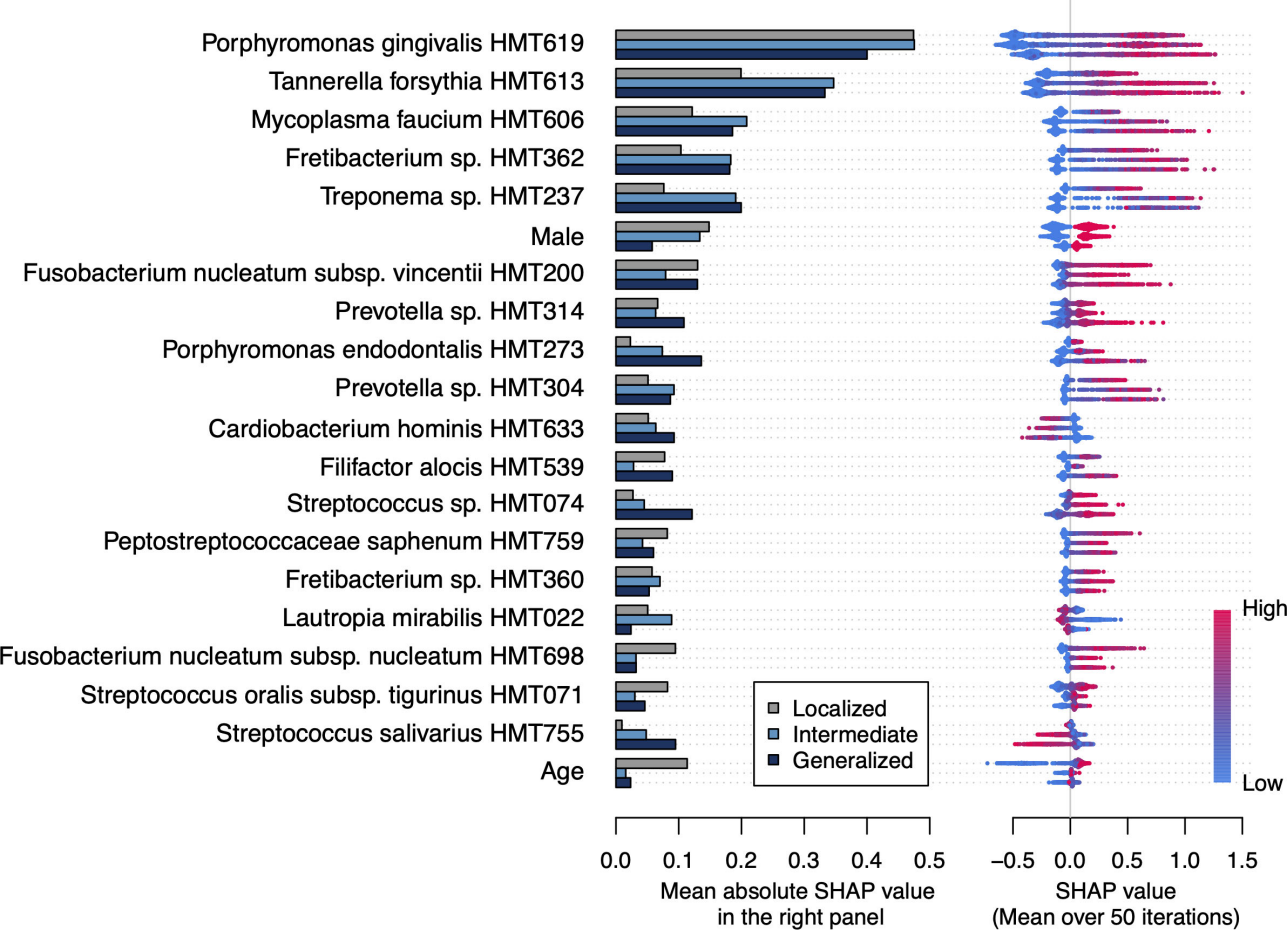
Important features for detecting periodontitis based on the biological definitions The bar plot shows the mean absolute Shapley additive explanation (SHAP) value on 50 iterations for each definition. A higher absolute SHAP value indicates a greater impact on the prediction. The features are ordered according to the mean absolute SHAP value among all definitions and the top 20 features are shown. Each dot represents each participant and the red color indicates that they are male, older, and have higher relative abundance of each taxon. The positive and negative values mean that the feature increases or decreases the probability of periodontitis for each participant, respectively.

To identify important features for detecting periodontitis, we calculated the mean SHAP value of each feature in the 50 models. **Figure 2** shows the top 20 most important features for detecting periodontitis based on the biological definitions (see **Supplementary Table 1** for the results by definition). *Porphromonas gingivalis* and *Tannerella forsythia* demonstrated the highest and second-highest SHAP values, respectively, for localized to generalized periodontitis. These were followed by sex, *Fusobacterium nucleatum subspecies vincentii*, and *Mycoplasma faucium* for the detection of localized periodontitis. For the detection of intermediate and generalized periodontitis, *M. faucium*, *Treponema* species HMT-237, and *Fretibacterium* species HMT-362 were particularly important, after *P. gingivalis* and *T. forsythia*. The relative abundances of *Cardiobacterium hominis*, *Lautropia mirabilis*, and *Streptococcus salivarius* negatively contributed to the detection of periodontitis.

## 4 Discussion

This study determined the salivary microbiota composition of 2,050 participants using 16S rRNA gene amplicon analysis, and verified its predictive performance in the detection of periodontitis using a machine learning approach. This approach demonstrated high performance in detecting periodontitis based on number of teeth with PD ≥4 mm, achieving an AUC value ≥0.80 not only in generalized cases but also in localized cases whose detection was limited in our previous study focusing on only subgingival-plaque specific bacteria in saliva (Ma et al., 2021). This result emphasizes the potential of whole salivary microbiota as a screening tool for periodontitis. Unlike dental examinations, which are time-consuming, invasive, and require technical tests, saliva collection is easy and noninvasive and does not require the expertise of dentists and hygienists. Furthermore, this type of salivary bacterial test is expected to contribute to the reassessment and improvement of oral health conditions. We believe that salivary microbiota has the potential to be used for extensive and non-burdensome screening that can estimate the necessity for visiting a dental office simply through collection and mailing of saliva.

For clinical application, it is necessary to set an appropriate cutoff value. Particularly, in the screening test, minimizing false negatives (individuals with periodontitis who test negative) is prioritized to reduce overlooking cases that require early intervention or urgent treatment. In detecting intermediate and generalized cases, the sensitivities were high (0.83 and 0.86, respectively), even when a cutoff value based on the Youden index was used, which considers a balance between sensitivity and specificity (Youden, 1950). Meanwhile, it seemed difficult to distinguish localized cases from healthy cases, as expected, and the sensitivity of localized cases (0.76) was lower than that of intermediate and generalized cases. When we recalculated the cutoff value based on an F_2_ score, which is a form of the F score calculated by sensitivity and precision and prioritizes sensitivity, the specificity declined to 0.60 but the sensitivity improved to 0.87 (Chinchor and Sundheim, 1993; Sasaki, 2007). In this case, false positives (individuals without periodontitis who test positive) may increase; however, false negatives will decrease. Such a trade-off and an appropriate cutoff value should be carefully considered in further investigations of independent populations.

During the prediction process, *P. gingivalis* and *T. forsythia* were identified as the most important features for the detection of periodontitis. They are classically well-known as the ”red complex” along with *Treponema denticola* because of their co-aggregation characteristics and strong association with periodontitis (Socransky et al., 1998; Holt and Ebersole, 2005). In addition, subgingival bacteria, such as *Fretibacterium* species HMT-362, *F. nucleatum*, *Porphyromonas endodontalis*, *Filifactor alocis*, and *Eubacterium saphenum*, have also been identified as important features (Pérez-Chaparro et al., 2014; Kageyama et al., 2017; Ma et al., 2021). These findings are consistent with our concept that the salivary microbiota contains bacteria shed from the subgingival space, which expands with the progression of periodontitis, and their abundance in the salivary microbiota can be used to detect periodontitis. This study also identified *M. faucium* as a critical feature, following *P. gingivalis* and *T. forsythia*. Although a few studies reported the detection of *M. faucium* from subgingival plaque in patients with periodontitis (Abusleme et al., 2013; Camelo-Castillo et al., 2015; Chen et al., 2018), *M. faucium* is considered as a member of microbiota on human oropharynx including palatine tonsils and might be involved in periodontitis in a manner different from subgingival bacteria (Freundt et al., 1974; Escapa et al., 2018). Further studies focusing on *M. faucium* may provide novel information to enhance predictive performance or understand periodontitis.

Aging is a known risk factor for periodontitis and was identified as the sixth important feature in detecting localized periodontitis. However, it was not listed in the top 20 features for intermediate and generalized periodontitis. These results suggest that an alteration in the bacterial composition of salivary microbiota by the progression of periodontitis occurs regardless of age and support the utility of salivary microbiota for detecting periodontitis.

In the present approach, the predictive performance was lower in detecting periodontitis based on the CDC and AAP case definitions compared with the biological definitions. This is partly because the former considers the clinical attachment loss (AL). For instance, the moderate definition includes cases with ≥2 interproximal sites with AL ≥4 mm. Although AL can be used to evaluate the degree of alveolar bone resorption and prior periodontitis, it is not necessarily accompanied by deep periodontal pockets. Therefore, there are many cases with no increase in subgingival bacteria in the oral cavity, and an accurate prediction may not be possible.

This study had some limitations. First, the species-level taxonomic assignment was based on the sequencing of the 16S rRNA gene V1–V2 regions. Although these regions are recommended for oral microbiota analysis because of their ability to discriminate oral streptococci from the V3–V4 regions (Wade and Prosdocimi, 2020), they might be insufficient to distinguish bacterial species with similar base sequences. Second, the present approach incurs costs for molecular analyses and sequencing. Although sequencing costs have drastically decreased over the past few decades, we should consider a cost-saving scheme such as simultaneous analysis of a large number of samples for social applications. Third, there is a need for further examination of the selection of machine learning models, input features, and outcome variables. Although we performed logistic regression analysis as a supplementary analysis, the predictive performance was lower than the present results (mean AUC values of 0.75, 0.76, and 0.77 for localized, intermediate, and generalized cases; **Supplementary Table 2**), suggesting the validity of a complex model, such as LightGBM. We further explored the community periodontal index (CPI) as an outcome, and the performances were mean AUC values of 0.77 and 0.78 for detecting participants with CPI scores ≥3 (with PD ≥4 mm) and 4 (with PD ≥6 mm), showing that screening results by CPI can also serve as a gold standard for future research (**Supplementary Table 2**). Fourth, this study included a dataset of Japanese adults, and the generalizability is limited. External validation using an independent dataset is required. Fifth, as the present results were based on a cross-sectional design, the potential utility of salivary microbiota for assessing the risk of future onset or progression of periodontitis should be further studied.

In conclusion, this study employed a machine learning approach using salivary microbiota data and highlighted the potential utility of salivary microbiota in the screening of periodontitis. Furthermore, some taxa have been identified as notable biomarkers for screening periodontitis. Further analyses assessing global generalizability, practicality, and costs would be required to support the development of a novel screening test based on salivary microbiota.

## Data Availability

The sequence data presented in the study are deposited in the DDBJ BioProject database, accession number PRJDB35955.

## References

[B1] Abusleme L. Dupuy A. K. Dutzan N. Silva N. Burleson J. A. Strausbaugh L. D.. (2013). The subgingival microbiome in health and periodontitis and its relationship with community biomass and inflammation. ISME J. 7, 1016–1025. doi: 10.1038/ismej.2012.174, PMID: 23303375 PMC3635234

[B2] Akiba T. Sano S. Yanase T. Ohta T. Koyama M. (2019). “Optuna: A next-generation hyperparameter optimization framework,” in Proceedings of the ACM SIGKDD International Conference on Knowledge Discovery and Data Mining. New York, NY, USA: Association for Computing Machinery. 2623–2631. doi: 10.1145/3292500.3330701

[B3] Angjelova A. Jovanova E. Polizzi A. Laganà L. Santonocito S. Ragusa R.. (2024). Impact of periodontitis on endothelial risk dysfunction and oxidative stress improvement in patients with cardiovascular disease. J. Clin. Med. 13, 3781. doi: 10.3390/jcm13133781, PMID: 38999345 PMC11242897

[B4] Belstrøm D. Sembler-Møller M. L. Grande M. A. Kirkby N. Cotton S. L. Paster B. J.. (2017). Microbial profile comparisons of saliva, pooled and site-specific subgingival samples in periodontitis patients. PloS One 12, e0182992–e0182992. doi: 10.1371/journal.pone.0182992, PMID: 28800622 PMC5553731

[B5] Bolyen E. Rideout J. R. Dillon M. R. Bokulich N. A. Abnet C. C. Al-Ghalith G. A.. (2019). Reproducible, interactive, scalable and extensible microbiome data science using QIIME 2. Nat. Biotechnol. 37, 852–857. doi: 10.1038/s41587-019-0209-9, PMID: 31341288 PMC7015180

[B6] Camelo-Castillo A. J. Mira A. Pico A. Nibali L. Henderson B. Donos N.. (2015). Subgingival microbiota in health compared to periodontitis and the influence of smoking. Front. Microbiol. 6. doi: 10.3389/fmicb.2015.00119, PMID: 25814980 PMC4356944

[B7] Chen W. P. Chang S. H. Tang C. Y. Liou M. L. Tsai S. J. J. Lin Y. L. (2018). Composition analysis and feature selection of the oral microbiota associated with periodontal disease. BioMed. Res. Int. 2018, 3130607. doi: 10.1155/2018/3130607, PMID: 30581850 PMC6276491

[B8] Chen T. Yu W. H. Izard J. Baranova O. V. Lakshmanan A. Dewhirst F. E. (2010). The Human Oral Microbiome Database: a web accessible resource for investigating oral microbe taxonomic and genomic information. Database 2010, baq013. doi: 10.1093/database/baq013, PMID: 20624719 PMC2911848

[B9] Chinchor N. Sundheim B. M. (1993). “MUC-5 evaluation metrics,” in Proceedings of the 5th Conference on Message Understanding. Stroudsburg, PA, USA: Association for Computational Linguistics. doi: 10.3115/1072017.1072026

[B10] Dolcezza S. Flores-Fraile J. Lobo-Galindo A. B. Montiel-Company J. M. Zubizarreta-Macho Á. (2024). Relationship between rheumatoid arthritis and periodontal disease-systematic review and meta-analysis. J. Clin. Med. 14, 10. doi: 10.3390/jcm14010010, PMID: 39797091 PMC11720692

[B11] Eke P. I. Page R. C. Wei L. Thornton-Evans G. Genco R. J. (2012). Update of the case definitions for population-based surveillance of periodontitis. J. Periodontol. 83, 1449–1454. doi: 10.1902/jop.2012.110664, PMID: 22420873 PMC6005373

[B12] Escapa I. F. Chen T. Huang Y. Gajare P. Dewhirst F. E. Lemon K. P. (2018). New insights into human nostril microbiome from the expanded human oral microbiome database (eHOMD): a resource for the microbiome of the human aerodigestive tract. mSystems 3, e00187-18. doi: 10.1128/msystems.00187-18, PMID: 30534599 PMC6280432

[B13] Freundt E. A. Taylor Robinson D. Purcell R. H. (1974). Proposal of Mycoplasma buccale nom. nov. and Mycoplasma faucium nom. nov. for Mycoplasma orale ‘types’ 2 and 3, respectively. Int. J. Syst. Bacteriol. 24, 252–255. doi: 10.1099/00207713-24-2-252

[B14] Hajishengallis G. (2014). Periodontitis: from microbial immune subversion to systemic inflammation. Nat. Rev. Immunol. 15, 30–44. doi: 10.1038/nri3785, PMID: 25534621 PMC4276050

[B15] Haririan H. Andrukhov O. Bertl K. Lettner S. Kierstein S. Moritz A.. (2014). Microbial analysis of subgingival plaque samples compared to that of whole saliva in patients with periodontitis. J. Periodontol. 85, 819–828. doi: 10.1902/jop.2013.130306, PMID: 24144271

[B16] Hata J. Ninomiya T. Hirakawa Y. Nagata M. Mukai N. Gotoh S.. (2013). Secular trends in cardiovascular disease and its risk factors in Japanese. Circulation 128, 1198–1205. doi: 10.1161/CIRCULATIONAHA.113.002424, PMID: 23902756

[B17] He J. Huang W. Pan Z. Cui H. Qi G. Zhou X.. (2012). Quantitative analysis of microbiota in saliva, supragingival, and subgingival plaque of Chinese adults with chronic periodontitis. Clin. Oral. Investig. 16, 1579–1588. doi: 10.1007/s00784-011-0654-4, PMID: 22169888

[B18] Holt S. C. Ebersole J. L. (2005). Porphyromonas gingivalis, Treponema denticola, and Tannerella forsythia: The ‘red complex’, a prototype polybacterial pathogenic consortium in periodontitis. Periodontology 2000 38, 72–122. doi: 10.1111/j.1600-0757.2005.00113.x, PMID: 15853938

[B19] Jung J.-S. Kook J.-K. Park S.-N. Lim Y. K. Choi G. H. Kim S.. (2024). Salivary microbiota reflecting changes in subgingival microbiota. Microbiol. Spectr. 12, e0103024. doi: 10.1128/spectrum.01030-24, PMID: 39365037 PMC11537074

[B20] Kageyama S. Furuta M. Takeshita T. Ma J. Asakawa M. Yamashita Y. (2022). High-level acquisition of maternal oral bacteria in formula-fed infant oral microbiota. mBio 13, e0345221. doi: 10.1128/mbio.03452-21, PMID: 35038919 PMC8764541

[B21] Kageyama S. Sakata S. Ma J. Asakawa M. Takeshita T. Furuta M.. (2023). High-resolution detection of translocation of oral bacteria to the gut. J. Dent. Res. 102, 752–758. doi: 10.1177/00220345231160747, PMID: 37204134 PMC10288163

[B22] Kageyama S. Takeshita T. Asakawa M. Shibata Y. Takeuchi K. Yamanaka W.. (2017). Relative abundance of total subgingival plaque-specific bacteria in salivary microbiota reflects the overall periodontal condition in patients with periodontitis. PloS One 12, e0174782. doi: 10.1371/journal.pone.0174782, PMID: 28369125 PMC5378373

[B23] Ke G. Meng Q. Finley T. Wang T. Chen W. Ma W.. (2017). LightGBM: A highly efficient gradient boosting decision tree. Adv. Neural Inf. Process. Syst. 30, 3147–3155.

[B24] Lamster I. B. Kaufman E. Grbic J. T. Winston L. J. Singer R. E. (2003). β-glucuronidase activity in saliva: relationship to clinical periodontal parameters. J. Periodontol. 74, 353–359. doi: 10.1902/jop.2003.74.3.353, PMID: 12710755

[B25] Liaw A. Liu C. Bartold M. Ivanovski S. Han P. (2023). Salivary histone deacetylase in periodontal disease: A cross-sectional pilot study. J. Periodontal Res. 58, 433–443. doi: 10.1111/jre.13104, PMID: 36717759

[B26] Lu X. Li P. Li J. Hu J. Tian R. (2023). Clinical diagnostic value of IL-14, 1L-16 and SAA in periodontitis. Clin. Oral. Investig. 27, 6627–6635. doi: 10.1007/s00784-023-05269-8, PMID: 37714977

[B27] Lundberg S. M. Erion G. Chen H. DeGrave A. Prutkin J. M. Nair B.. (2020). From local explanations to global understanding with explainable AI for trees. Nat. Mach. Intell. 2, 56–67. doi: 10.1038/s42256-019-0138-9, PMID: 32607472 PMC7326367

[B28] Ma J. Kageyama S. Takeshita T. Shibata Y. Furuta M. Asakawa M.. (2021). Clinical utility of subgingival plaque-specific bacteria in salivary microbiota for detecting periodontitis. PloS One 16, e0253502. doi: 10.1371/journal.pone.0253502, PMID: 34170942 PMC8232462

[B29] Maeng Y.-J. Kim B.-R. Jung H.-I. Jung U.-W. Kim H. E. Kim B.-I. (2016). Diagnostic accuracy of a combination of salivary hemoglobin levels, self-report questionnaires, and age in periodontitis screening. J. Periodontal Implant Sci 46, 10–10. doi: 10.5051/jpis.2016.46.1.10, PMID: 26937290 PMC4771833

[B30] Nomura Y. Tamaki Y. Tanaka T. Arakawa H. Tsurumoto A. Kirimura K.. (2006). Screening of periodontitis with salivary enzyme tests. J. Oral. Sci. 48, 177–183. doi: 10.2334/josnusd.48.177, PMID: 17220614

[B31] Papapanou P. N. Sanz M. Buduneli N. Dietrich T. Feres M. Fine D. H.. (2018). Periodontitis: Consensus report of workgroup 2 of the 2017 World Workshop on the Classification of Periodontal and Peri-Implant Diseases and Conditions: Classification and case definitions for periodontitis. J. Clin. Periodontol. 45 Suppl 20, S162–S170. doi: 10.1111/jcpe.12946, PMID: 29926490

[B32] Pedregosa F. Varoquaux G. Gramfort A. Michel V. Thirion B. Grisel O.. (2011). Scikit-learn: machine learning in python. J. Mach. Learn. Res. 12, 2825–2830. doi: 10.5555/1953048.2078195, PMID: 34820480

[B33] Pérez-Chaparro P. J. Gonçalves C. Figueiredo L. C. Faveri M. Lobão E. Tamashiro N.. (2014). Newly identified pathogens associated with periodontitis: a systematic review. J. Dent. Res. 93, 846–858. doi: 10.1177/0022034514542468, PMID: 25074492 PMC4541103

[B34] Pihlstrom B. L. Michalowicz B. S. Johnson N. W. (2005). Periodontal diseases. Lancet 366, 1809–1820. doi: 10.1016/S0140-6736(05)67728-8, PMID: 16298220

[B35] Rognes T. Flouri T. Nichols B. Quince C. Mahé F. (2016). VSEARCH: A versatile open source tool for metagenomics. PeerJ 2016, e2584–e2584. doi: 10.7717/PEERJ.2584, PMID: 27781170 PMC5075697

[B36] Sasaki Y. (2007). The truth of the F-measure. Teach tutor mater 1, 1–5.

[B37] Shimazaki Y. Akifusa S. Takeshita T. Shibata Y. Doi Y. Hata J.. (2011). Effectiveness of the salivary occult blood test as a screening method for periodontal status. J. Periodontol. 82, 581–587. doi: 10.1902/jop.2010.100304, PMID: 21043793

[B38] Socransky S. S. Haffajee A. D. Cugini M. A. Smith C. Kent R. L. (1998). Microbial complexes in subgingival plaque. J. Clin. Periodontol. 25, 134–144. doi: 10.1111/j.1600-051X.1998.tb02419.x, PMID: 9495612

[B39] Takeshita T. Kageyama S. Furuta M. Tsuboi H. Takeuchi K. Shibata Y.. (2016). Bacterial diversity in saliva and oral health-related conditions: the Hisayama Study. Sci. Rep. 6, 22164. doi: 10.1038/srep22164, PMID: 26907866 PMC4764907

[B40] Umeda M. Contreras A. Chen C. Bakker I. Slots J. (1998). The utility of whole saliva to detect the oral presence of periodontopathic bacteria. J. periodontology 69, 828–833. doi: 10.1902/jop.1998.69.7.828, PMID: 9706862

[B41] Wade W. G. Prosdocimi E. M. (2020). Profiling of oral bacterial communities. J. Dent. Res. 99, 621–629. doi: 10.1177/0022034520914594, PMID: 32286907 PMC7243418

[B42] Wu Y. C. Ning L. Tu Y. K. Huang C. P. Huang N. T. Chen Y. F.. (2018). Salivary biomarker combination prediction model for the diagnosis of periodontitis in a Taiwanese population. J. Formos. Med. Assoc. 117, 841–848. doi: 10.1016/j.jfma.2017.10.004, PMID: 29129647

[B43] Youden W. J. (1950). Index for rating diagnostic tests. Cancer 3, 32–35. doi: 10.1002/1097-0142(1950)3:1<32::AID-CNCR2820030106>3.0.CO;2-3 15405679

