# Supplementary material for: Performance of salivary microbiota in detecting periodontitis using a machine learning approach
Source: Front Cell Infect Microbiol. 2025 Sep 18;15:1631798. doi: 10.3389/fcimb.2025.1631798 (PMC12488677; doi:10.3389/fcimb.2025.1631798)
Supplement: Supplementary file 2 [file Table2.docx]

**Supplemental table 2. Predictive performance in supplementary analyses**

| **Outcome** | **Algorithm** | **AUC** | **Sensitivity** | **Specificity** |
| --- | --- | --- | --- | --- |
| Biological definitions |  |  |  |  |
| Localized | Logistic regression | 0.75 (0.74–0.75) | 0.65 (0.62–0.68) | 0.74 (0.71–0.77) |
| Intermediate |  | 0.76 (0.75–0.77) | 0.71 (0.68–0.74) | 0.74 (0.71–0.76) |
| Generalized |  | 0.77 (0.75–0.78) | 0.75 (0.72–0.79) | 0.72 (0.69–0.76) |
| CPI score |  |  |  |  |
| Score ≥3 | LightGBM | 0.77 (0.76–0.77) | 0.70 (0.68–0.72) | 0.72 (0.70–0.74) |
| Score 4 |  | 0.78 (0.77–0.79) | 0.74 (0.72–0.76) | 0.72 (0.69–0.74) |

Data are presented as mean values (95% confidence intervals) for 50 iterations. As the biological definitions, presence of ≥2, ≥4, and ≥6 teeth with probing depth ≥4 mm was defined as localized, intermediate, and generalized periodontitis. Sensitivity and specificity were determined using cut-off values based on the Youden index. Logistic regression (penalty=l1 and solver=saga) was performed along with a five-fold stratified cross-validation. The CPI score for each individual was determined as the highest score among 10 teeth (two molars and the upper right and lower left central incisors) examined using a WHO probe, with PD 4–5 mm scored as 3 and PD ≥6 mm scored as 4.

AUC, area under the receiver operating characteristic curve; CPI, community periodontal index; PD, probing depth.
